# Non-canonical translation initiation of the spliced mRNA encoding the human T-cell leukemia virus type 1 basic leucine zipper protein

**DOI:** 10.1093/nar/gky1249

**Published:** 2018-12-19

**Authors:** C Joaquín Cáceres, Jenniffer Angulo, Fernando Lowy, Nataly Contreras, Beth Walters, Eduardo Olivares, Delphine Allouche, Anne Merviel, Karla Pino, Bruno Sargueil, Sunnie R Thompson, Marcelo López-Lastra

**Affiliations:** 1 Departamento de Enfermedades Infecciosas e Inmunología Pediátrica, Laboratorio de Virología Molecular, Instituto Milenio de Inmunología e Inmunoterapia, Centro de Investigaciones Médicas, Escuela de Medicina, Pontificia Universidad Católica de Chile, Marcoleta 391, Santiago, Chile; 2 Department of Microbiology, University of Alabama at Birmingham, Birmingham AL 35294, USA; 3 CNRS UMR 8015, Laboratoire de cristallographie et RMN Biologique, Université Paris Descartes, 4 avenue de l’Observatoire, 75270 Paris Cedex 06, France

*Nucleic Acids Research*, 2018, Vol 46, No. 20, Pages 11030–11047, https://doi.org/10.1093/nar/gky802

This article has been updated to correct a grant number listed in the paper.

